# A Spatio-Temporal Joint Diagnosis Framework for Bearing Faults via Graph Convolution and Attention-Enhanced Bidirectional Gated Networks

**DOI:** 10.3390/s25133908

**Published:** 2025-06-23

**Authors:** Zhiguo Xiao, Xinyao Cao, Huihui Hao, Siwen Liang, Junli Liu, Dongni Li

**Affiliations:** 1 School of Computer Science & Technology, Beijing Institute of Technology, Beijing 100811, China; 3220215169@bit.edu.cn; 2 College of Computer Science and Technology, Changchun University, Changchun 130022, China; 241501503@mails.ccu.edu.cn (X.C.); 241503554@mails.ccu.edu.cn (J.L.); 3 National Key Laboratory of Special Vehicle Design and Manufacturing Integration Technology, Baotou 014000, China; haohui54670421@126.com (H.H.); lsw617@126.com (S.L.)

**Keywords:** bearing fault diagnosis, graph convolutional networks, bidirectional gated recurrent units, spatio-temporal features, attention mechanism

## Abstract

In recent years, Academia and industry have conducted extensive and in-depth research on bearing-fault-diagnosis technology. However, the current modeling of time–space coupling characteristics in rolling bearing fault diagnosis remains inadequate, and the integration of multi-modal correlations requires further improvement. To address these challenges, this paper proposes a joint diagnosis framework integrating graph convolutional networks (GCNs) with attention-enhanced bidirectional gated recurrent units (BiGRUs). The proposed framework first constructs an improved K-nearest neighbor-based spatio-temporal graph to enhance multidimensional spatial–temporal feature modeling through GCN-based spatial feature extraction. Subsequently, we design an end-to-end spatio-temporal joint learning architecture by implementing a global attention-enhanced BiGRU temporal modeling module. This architecture achieves the deep fusion of spatio-temporal features through the graph-structural transformation of vibration signals and a feature cascading strategy, thereby improving overall model performance. The experiment demonstrated a classification accuracy of 97.08% on three public datasets including CWRU, verifying that this method decouples bearing signals through dynamic spatial topological modeling, effectively combines multi-scale spatiotemporal features for representation, and accurately captures the impact characteristics of bearing faults.

## 1. Introduction

As core rotating components in mechanical systems, bearings perform critical functions including supporting shaft systems, reducing friction losses, transmitting loads, and ensuring motion precision. Their fault diagnosis is essential for safeguarding equipment health [1]. Based on structural differences, bearings are categorized into rolling bearings and sliding bearings, which are widely utilized in modern industrial fields such as manufacturing equipment, transportation, and energy power systems. Due to their crucial role in mechanical systems, the performance of bearings directly determines the reliability, efficiency, and lifespan of the mechanical systems. They hold an important position in the industrial field [2,3]. As key components in rotating machinery, the health status of rolling bearings has a decisive impact on the normal operation of entire mechanical systems [4]. Bearings operating under complex conditions over long-term periods inevitably experience performance degradation or faults. Therefore, developing advanced fault-diagnosis methodologies holds significant engineering application value [5,6].

In the field of bearing fault diagnosis, traditional methodologies primarily rely on manual feature extraction and threshold setting. However, practical engineering environments often present complex challenges such as non-stationary vibration signals, multi-source noise interference, and coupled fault modes. These factors significantly constrain the hidden information extraction capability of conventional approaches when processing high-dimensional signals, thereby limiting their feature representation capacity [7,8]. In order to overcome the limitations of traditional bearing-fault-diagnosis methods, the previous related research mainly adopted two technical paradigms. Firstly, physics model-based methods analyze fault mechanisms through dynamic equations, exemplified by Hertz contact theory [9]. Nevertheless, this approach exhibits poor generalization performance under variable loads and multi-fault coupling conditions due to its heavy dependence on precise prior knowledge, making it difficult to satisfy diagnostic requirements across diverse operational scenarios [10]. Secondly, data-driven approaches have emerged as alternative solutions. Traditional shallow models such as support vector machines (SVM) [11] and random forests [12] require manual feature engineering to extract discriminative fault characteristics. In practical applications, noise interference severely degrades the model robustness, compromising the diagnostic accuracy. Deep learning architectures including convolutional neural networks (CNNs) [13], long short-term memory networks (LSTMs) [14], and Transformer models [15] demonstrate automated feature extraction capabilities but exhibit structural performance trade-offs. Specifically, CNNs excel at capturing local features while neglecting long-range dependencies, whereas Transformers prioritize global pattern modeling at the expense of local dynamic information capture [16,17,18]. A novel predictive approach that integrates physics-based models with deep learning is emerging. This approach significantly enhances the deep analytical capabilities and predictive reliability of bearing fault detection by synergistically combining the advantages of mechanistic modeling and data-driven techniques [19].

Although the aforementioned research methodologies have introduced innovative perspectives to the field of fault diagnosis, several critical challenges persist in practical engineering implementations. First, significant limitations exist in the decoupling of spatio-temporal features: real-time rolling bearing data acquired through sensor arrays inherently contains spatial topological relationships and temporal dynamic characteristics, yet current approaches fail to achieve the efficient joint modeling of these multidimensional attributes [20]. Second, pronounced multi-scale feature separation phenomena occur during fault analysis, where device-specific local fault information reflected by local resonance features proves difficult to integrate with global degradation trends that characterize the overall operational status. Third, an inherent trade-off persists between model efficiency and robustness—complex architectures theoretically enable richer fault feature capture through substantial computational complexity, but this hinders their real-time implementation in online diagnostic applications.

This paper proposes a parallel fusion network model consisting of a graph convolutional network modeled by improved KNN and a bidirectional gated recurrent unit integrated with a global attention mechanism. Experimental validation on public bearing datasets demonstrates its superior fault-identification performance over mainstream models in terms of accuracy, computational efficiency, and noise robustness. The key contributions of the proposed methodology include the following:

(1) We design a dynamic topological modeling framework for rolling bearing data. By integrating a modified K-nearest neighbor (KNN) algorithm with graph convolutional networks (GCNs), this approach systematically mines and models spatio-temporal coupled features, effectively addressing the spatial–temporal modeling decoupling issue inherent to conventional methodologies.

(2) For temporal feature extraction, this study integrates a lightweight global attention mechanism to enable adaptive noise suppression gating. This approach dynamically filters interference signals while precisely capturing both the short-term and long-term dependencies of fault-induced impacts, thereby significantly enhancing the model’s robustness in complex noise environments and ensuring reliable and stable diagnostic outcomes.

(3) The network architecture employs a dual-stream parallel framework that synchronously extracts spatial topological features and temporal sequence characteristics through graph convolutional networks (GCNs) and bidirectional gated recurrent units (BiGRUs), respectively. To achieve effective multi-scale representation fusion, this work innovatively introduces a gated fusion mechanism that adaptively integrates heterogeneous features via a dynamic weight adjustment strategy.

This paper addresses the core challenges in rolling bearing fault diagnosis regarding the difficulty of spatiotemporal feature decoupling and insufficient multimodal information fusion by innovatively constructing a dual-stream parallel architecture integrating graph convolutional networks with attention-enhanced temporal models. The remainder of this paper is structured as follows: Section 2 reviews related research works on GRUs and attention mechanisms. Section 3 provides a detailed exposition of the proposed method, including the modeling methodology, BRGU channels enhanced with global attention mechanisms, and the feature fusion framework. Section 4 presents experimental setups, evaluation metrics, results, and analyses. Finally, Section 5 summarizes the paper while discussing its limitations and future research directions.

## 2. Related Work

### 2.1. Dynamic Graph Structure Modeling

Recent years have witnessed significant advancements in deep-learning-based bearing fault diagnosis methodologies. However, existing research still faces limitations in spatio-temporal feature joint modeling, dynamic graph structure optimization, and attention mechanism design. Traditional methodologies primarily rely on signal processing techniques combined with shallow machine learning models. For instance, Peng et al. [21] highlighted that time–domain, frequency–domain, and hybrid-domain features—such as root mean square (RMS) values and wavelet packet decomposition coefficients—are conventionally extracted and subsequently classified via K-nearest neighbors (KNNs) or support vector machines (SVMs). Liu and Weng [22] proposed a workflow where the integration of wavelet packet decomposition and SVM effectively identifies resonance frequency bands, yet this approach heavily depends on manual feature engineering and struggles to capture nonlinear spatio-temporal correlations under complex operational conditions. Khan et al. [23] further emphasized that while multi-modal signal analysis (e.g., vibration and current signals) improves robustness, it faces challenges in feature redundancy and limited cross-domain generalization. These approaches remain vulnerable to noise interference in dynamic industrial scenarios and fail to adaptively extract deep semantic features from high-dimensional data.

Graph convolutional networks (GCNs) have emerged as a research hotspot by modeling complex relationships between data through graph structures. Wang et al. [24] proposed a multi-layer GCN (AE-MSGCN) that constructs multi-metric graphs using KNN and cosine similarity, enhancing node representations through neighbor information aggregation. This approach outperformed traditional models in bearing diagnostics. Zhang et al. [25] further developed a multi-source cross-domain fault diagnosis framework called the Graph Attention Convolutional Neural Network (GACNN), which constructs time–frequency graphs and employs attention-driven domain adaptation to align cross-domain feature distributions, improving the diagnostic accuracy under variable operating conditions. However, existing GCN methods predominantly rely on fixed adjacency matrices, neglecting temporal dynamics and suffering from theoretically unsupported self-loop edge definitions and weight allocation strategies. To address these limitations, this study proposes an enhanced strategy targeting bearing signal sequence modeling requirements: improved K-nearest neighbor graph construction methodology, integration of spatio-temporal encoding, adaptive weighted graph generation, and hyperparameter optimization through GNN cross-validation.

### 2.2. Research and Principles Related to GRU Units and Global Attention

**Figure 1:**
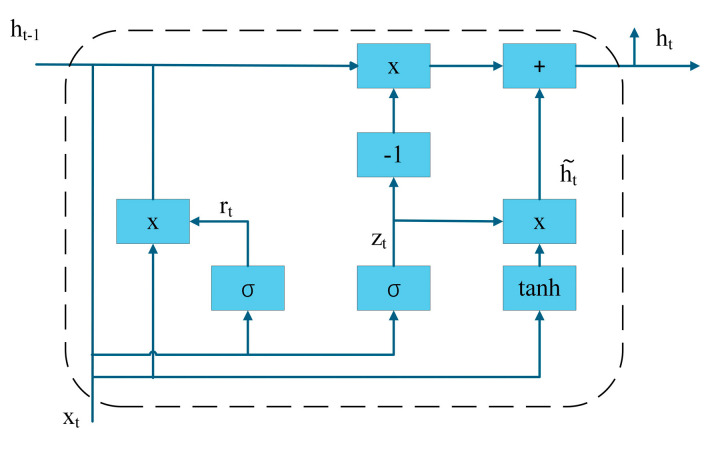
GRU unit.

Previous studies have predominantly employed traditional deep learning approaches such as Transformers for bearing fault detection [26]. However, these methods inherently suffer from temporal correlation information loss due to their reliance on manual feature engineering and sequence format conversion. Recurrent neural networks (RNNs), particularly their advanced variants Long Short-Term Memory (LSTM) and gated recurrent units (GRUs), have gained attention for their native temporal modeling capabilities through chain-structured architectures. Among these, GRU demonstrate superior performance by integrating an update gate mechanism that maintains long-term dependency modeling capability while significantly reducing computational complexity [27]. The internal architecture of the GRU neural network is illustrated in Figure 1.

In the diagram, $Z_{t}$ and $r_{t}$ denote the update gate and reset gate, respectively, $\tilde{h_{t}}$ represents the candidate hidden state at time t, $h_{t-1}$ is the input from the previous time step, and $h_{t}$ is the output. Specifically, the update gate $Z_{t}$ is calculated via Equation (1), the reset gate $r_{t}$ follows Equation (2), the candidate hidden state $\;\tilde{h_{t}}$ is derived using Equation (3), and the final output $\;h_{t}$ is computed through Equation (4).

(1) $$Z_{t}=\sigma \left(W_{xz}x_{t}+W_{hz}h_{t-1}+b_{z}\right)$$

(2) $$r_{t}=\sigma (W_{xx}x_{t}+W_{hr}h_{t-1}+b_{r})$$

(3) $$\tilde{h_{t}}=\tanh(W_{xh}x_{t}+r_{t}\cdot h_{t-1}W_{hh}+b_{h})$$

(4) $$h_{t}=(1-z_{t})\cdot \tilde{h_{t}}+z_{t}\cdot h_{t-1}$$

Here, W denotes the weight matrix between the update gate and reset gate, σ represents the sigmoid activation function, and $r_{t}$ is the reset gate with values ranging between 0 and 1. When $r_{t}\;$= 0, it indicates the complete forgetting of previous temporal information transmitted from the prior time step; b denotes the bias vector.

**Figure 2:**
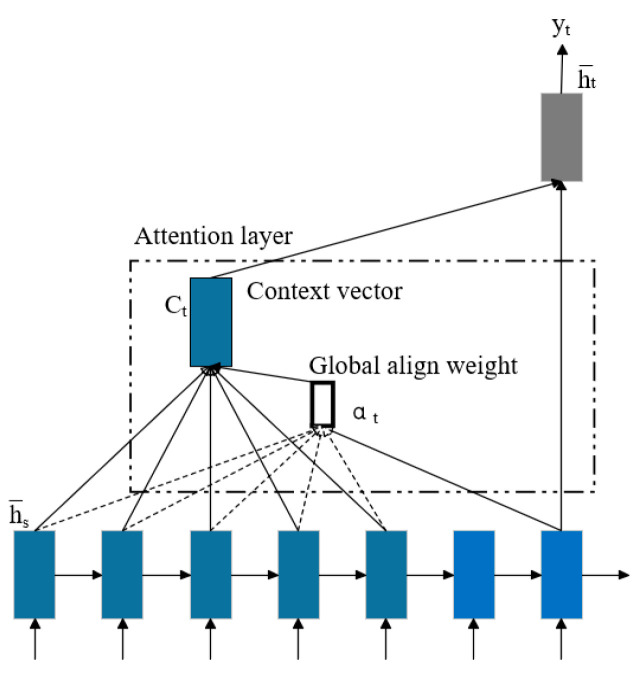
Global attention mechanism.

In temporal modeling tasks, the attention mechanism significantly enhances the model’s ability to capture critical features by simulating the selective attention mechanism in human cognition [28,29]. The global attention model computes the context vector $c_{t}$ by incorporating all hidden states of the encoder during derivation. The architecture of the global attention mechanism is illustrated in Figure 2.

Among them, by comparing the decoded target hidden state $h_{t}$ at time t with the source hidden state sequence $h_{last}$ output by the encoder (where s=1,2,...,S,S is the length of the source sequence), an aligned weight context vector **c** of the same length as the source sequence can be dynamically generated. The mathematical form of the derivation process is shown in Equation (5).

(5) $$c=\sum_{t=1}^{S}\alpha_{t}h_{t}=\frac{\exp(V\tanh(W\left[h_{last}+h_{t}\right]))}{\sum_{t^{\prime }=1}^{T}\exp(e_{t^{\prime }})}$$

Here, ***V*** and ***W*** represent the weight coefficient matrices that need to be trained in the network, and $h_{last}$ is the hidden state of the last layer. The model adaptively allocates attention weights at each decoding time step t, and finally obtains the global context vector **c** by weighted summation of all source hidden states.

Compared with a unidirectional GRU, the proposed BiGRU architecture considers data variation patterns through independent forward and backward hidden layers, enabling superior sequence feature extraction. The bidirectional context capture enhances the temporal modeling capabilities. He et al. [30] validated the superiority of BiGRU-BP in runoff prediction, while Sun et al. [31] optimized BiGRU input representations by integrating Inception modules. This work concatenates hidden layers with output states at BiGRU terminals and implements feature dynamic weighting through a global attention mechanism. Compared with the multimodal fusion DNN (DGFFDNN) proposed by Zhou et al. [32], our approach emphasizes spatio-temporal feature complementarity. Attention mechanisms significantly improve time series prediction and diagnostic performance. Qin et al. [33] introduced the DA-RNN model, which employs input attention for feature selection and temporal attention for long-term dependency capture, providing novel insights for sequential modeling. Feng [34] further incorporated switch-gated LSTMs to optimize attention weight allocation in multivariate time series. Yang et al. [35] combined graph neural networks (GNNs) with attention mechanisms for one-shot fault propagation reasoning. This study proposes a global attention mechanism that dynamically weights key time steps, effectively suppressing feature extraction redundancy in BiGRU-based fault diagnosis.

Furthermore, spatio-temporal fusion strategies have become critical for improving the diagnostic accuracy. Zhang et al. [36] proposed the PG-STF framework, which combines node spatial encoding with dual-supervised training by leveraging prior knowledge to construct physically correlated graphs, significantly enhancing diagnostic reliability in chemical processes. Xing et al. [37] developed a sliding-window KNN hybrid model (STHM) that weights spatio-temporal statistics via the CUSUM algorithm. However, this approach lacks deep learning integration and still exhibits significant limitations in nonlinear feature representation and complex pattern recognition. In contrast, this work innovatively introduces a dynamic feature fusion framework based on gated recurrent units. By constructing channel-wise attention weights through learnable sigmoid activation functions, the method achieves the adaptive weighted fusion of multi-channel parallel features.

## 3. The Proposed Method

### 3.1. Improved KNN-Based Spatiotemporal Modeling of Rolling Bearing Data

**Figure 3:**
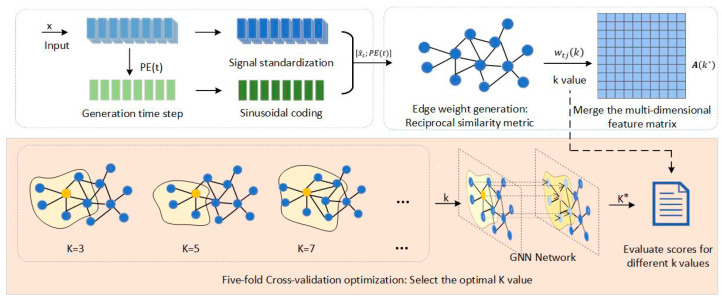
Construct the spatio-temporal feature encoder based on KNN.

In response to the demand for the model structure mining ability caused by the spatio-temporal coupling characteristics of vibration signals in bearing fault diagnosis, this paper improves the graph structure modeling method based on the K-nearest neighbor algorithm, breaks through the bottlenecks of traditional KNN, such as the fault of similarity measurement in high-dimensional heterogeneous feature Spaces, insufficient adaptability to dynamic working conditions, and excessive cost of global parameter optimization, and innovatively proposes a multi-dimensional feature enhancement strategy: (1) construct a spatio-temporal fusion feature coding system; (2) design an adaptive generation algorithm for weighted graph structures; (3) establish a K-fold cross-validation hyperparameter optimization method based on graph neural networks. The specific model diagram is shown in Figure 3.

The proposed graph topology modeling methodology follows these sequential procedures: first, the original time-series signals are transformed into high-dimensional spatio-temporal feature representations using sinusoidal encoding technology; subsequently, a distance measurement model is established based on the reciprocal of node correlation coefficients to quantify spatial correlations, which serves as the edge weight parameters in the adjacency matrix; based on this framework, the optimal neighborhood parameter K is selected through a five-fold cross-validation strategy; finally, the spatio-temporal characteristic graph topology is constructed by integrating the node connection sequence determined through the K-nearest neighbor (KNN) algorithm.

First, standardization preprocessing is implemented: the original signal $x={\left[x_{1},x_{2},\ldots ,x_{T}\right]}^{⊤}\in R^{T}$ and label $y=\left\{0,1\right\}$ are subjected to feature standardization using Z-score normalization. The standardized signal ${\hat{x}}_{t}$ is calculated using Equation (6).

(6) $${\hat{x}}_{t}=\frac{x_{t}-\mu }{\sigma },\;t=1,2,\ldots ,T$$

The mean value μ of the time series is calculated using Equation (7), determined by computing the arithmetic mean of all original signals $x_{t}$ across time steps t; the standard deviation σ is calculated using Equation (8), specifically by summing the squared deviations of each time-step signal from the mean, dividing by the total time steps T, and taking the square root. These computations are formally expressed in Equations (7) and (8):

(7) $$\mu =\frac{1}{T}\sum_{t=1}^{T}x_{t}$$

(8) $$\sigma =\sqrt{\frac{1}{T}\sum_{t=1}^{T}{\left(x_{t}-\mu \right)}^{2}}$$

A 32-dimensional sinusoidal positional encoding scheme is applied to time-step t, generating positional embedding vectors for time-step indices t using Equation (9). This encoding approach constructs periodic feature representations by designing sinusoidal basis functions with different frequency parameters across even dimensions, effectively capturing dynamic evolution patterns in time series and enhancing the model’s temporal position awareness capability.

(9) $$PE\left(t,2i\right)=\sin\left(\frac{t}{10000^{2i/32}}\right),PE\left(t,2i+1\right)=\cos\left(\frac{t}{10000^{2i/32}}\right)$$

where i=0,1,…,15 represents the discretized configuration of sinusoidal encoding frequency parameters. Based on this design, spatio-temporal node features are constructed. Specifically, the node feature at time step t is represented as $X_{t}=\left[{\hat{x}}_{t};PE(t)\right]\in R^{33}$, where ${\hat{x}}_{t}$ denotes the standardized original signal feature, and $PE(t)\in R^{32}$ is the 32-dimensional positional encoding vector generated via Equation (9).

Second, an improved K-nearest neighbor (KNN) algorithm is employed to construct a dynamic graph topology. Specifically, a candidate neighborhood parameter set $K=\left\{3,5,7,11,13\right\}$ is first defined, and for each k∈K, the graph structure is generated through the following sequential steps: (1) a node similarity matrix is constructed using Euclidean distance measurements, as formulated in Equation (10); (2) a *k*-nearest neighbor connection relationship is established for each node t, generating the corresponding adjacency matrix A(k); (3) edge weights $w_{tj}\left(k\right)$ are calculated based on the reciprocal of node correlation coefficients.

(10) $$N_{k}\left(t\right)={Top}_{k}\left(arg{min}_{j\neq t}{\left\|x_{t}-x_{j}\right\|}_{2}\right)$$

For the k-nearest neighbor set $N_{k}\left(t\right)$ of node t, the edge weight $w_{tj}\left(k\right)$ between node t and its neighboring node j is calculated using Equation (11). Under traditional Euclidean distance measurements, physical distances between nodes exhibit negative correlations with similarity metrics. By employing a reciprocal similarity measure synergistically optimized with graph convolutional network (GCN) propagation characteristics, the model achieves enhanced local topological structure perception capability while maintaining computational efficiency.

(11) $$w_{tj}(k)=\frac{1}{{\left\|x_{t}-x_{j}\right\|}_{2}+\epsilon },\epsilon =10^{-5}$$

where ϵ is a minimal constant added to prevent division by zero in reciprocal calculations. The optimal *K* value is selected using a five-fold cross-validation methodology: for each candidate *K* value, it serves as an input parameter to a two-layer graph convolutional network (GCN) for model training and performance evaluations. By comparing validation set performances across different *K* values, the optimal parameter *K* is ultimately determined. The mathematical formulation of the evaluation model is defined through a recursive computation in Equation (12).

(12) $$H^{\left(l\right)}(k)=\sigma ({\tilde{D}(k)}^{-1/2}\tilde{A}(k){\tilde{D}(k)}^{-1/2}H^{\left(l-1\right)}W^{\left(l\right)})$$

where $H^{\left(l\right)}$ denotes the hidden state at layer l, σ is the activation function, $\tilde{A}=A\left(k\right)+I$ with I as the identity matrix, $\tilde{D}$ is the degree matrix, and $W^{\left(l\right)}$ represents the weight matrix at layer l. The classification accuracy Acc(*k*) for each candidate k is computed through five-fold cross-validation. The optimal neighborhood parameter is finally selected as $k^{*}=\arg\max Acc(k)$. Based on this optimal parameter, the final adjacency matrix $A\left(k\right)$ is reconstructed using Equation (13), completing the parametric modeling of the graph topology.

(13) $$A\left(k^{*}\right)=\left\{\begin{array}{c} \frac{1}{{\left\|x_{t}-x_{j}\right\|}_{2}+\epsilon },j\in N_{k^{*}}\left(t\right)\\ 0 \end{array}\right.$$

### 3.2. The Main Procedure of the ST-GABG Diagnostic Model

**Figure 4:**
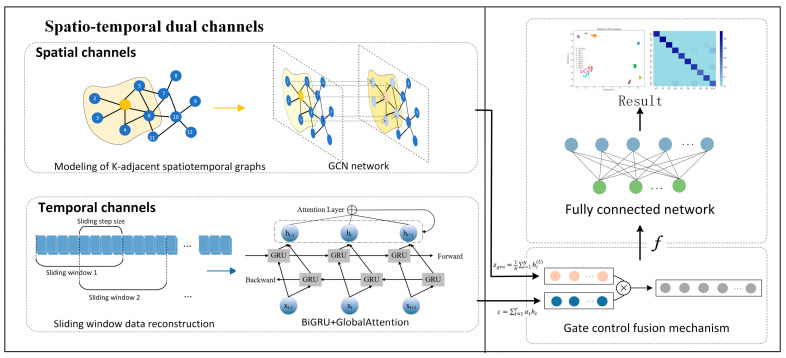
The overall process of the ST-GABG diagnostic model.

To address the insufficiency of traditional bearing-fault-diagnosis methods in spatio-temporal feature joint modeling, this study proposes a cross-modal dual-channel diagnostic framework based on graph convolutional-bidirectional gated recurrent units (GCN-BiGRU), termed ST-GABG (Figure 4). By integrating a dynamic K-nearest neighbor algorithm to construct sensor correlation topologies and leveraging GCN for multi-order neighborhood information aggregation, the framework overcomes fixed receptive field limitations. A BiGRU-global attention fusion architecture is designed to strengthen periodic impact signal capture through bidirectional temporal modeling, while dynamically focusing on critical temporal nodes using attention weights. Additionally, a parameter-efficient gated fusion mechanism adaptively regulates interaction weights between spatial topology features and temporal evolution features, achieving synergistic representation of compound fault characteristics.

The specific implementation process of the ST-GABG diagnostic model is as follows:

(1) Graph data modeling and feature extraction network for rolling bearing data. Based on the dynamic graph topology modeling method proposed in Section 3.1, the adjacency matrix $A\left(k\right)$ incorporating node feature information is constructed to complete data relationship representation. Subsequently, the graph-structured data is fed into a three-layer graph convolutional network (GCN) module for feature extraction, followed by average pooling that performs row-wise mean operations on the adjacency matrix using Equation (14). This process simultaneously smooths noise interference while enhancing global feature distribution characteristics.

(14) $$z_{gcn}=\frac{1}{N}\sum_{i=1}^{N}h_{i}^{\left(L\right)}\in R^{256}$$

where $z_{gcn}$ denotes the graph-level feature vector output by the GCN, which serves as input for subsequent tasks. N represents the total number of nodes in the graph, and $h_{i}^{\left(L\right)}$ indicates the hidden state of the *i*-th node at the *L*-th layer of the GCN. The final feature vector is mapped through a fully connected layer to generate the ultimate output feature $z_{gcn}$ with dimensions (batch_size,256).

**Figure 5:**
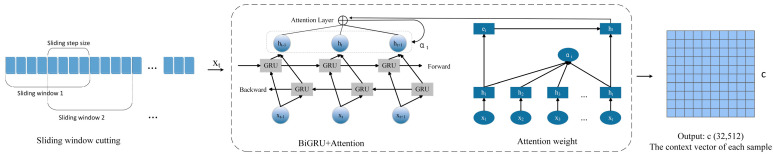
BiGRU and attention fusion architecture.

(2) Temporal Data Feature Extraction Network Based on Bidirectional Gated Recurrent Units. This module reconstructs the original time-series data, transforming it into a structured training dataset with temporal characteristics. Subsequently, a bidirectional gated recurrent unit (BiGRU) network architecture is constructed to achieve deep feature extraction from input sequences through a synergistic mechanism of forward and backward recursive computation. The network structure is illustrated in Figure 5.

To address gradient instability and feature extraction challenges in long-sequence signal processing, this module employs a sliding window reconstruction strategy. The original 1024-dimensional time-series signals are partitioned into 32 consecutive temporal windows, each containing 32-dimensional feature points, thereby constructing a 32 × 32 temporal feature tensor.

Subsequently, a bidirectional gated recurrent unit (BiGRU) network architecture is constructed. Through collaborative operations of forward and backward recurrent neurons, this architecture performs deep temporal feature extraction on input sequences to capture bidirectional temporal dependencies in the data. Specifically, the input data $x_{t}$ is fed into both the forward and backward hidden layers. The forward and backward units receive the input along with the forward hidden state ${\vec{h}}_{t-1}$ and backward hidden state ${\overset{\leftarrow }{h}}_{t-1}$ at time *t*−1. The current forward hidden state ${\vec{h}}_{t}$ and backward hidden state ${\overset{\leftarrow }{h}}_{t}$ at time *t* are then computed through GRU units. The final hidden state $h_{t}$ is derived according to Equation (15).

(15) $$h_{t}=W_{{\vec{h}}_{t}}{\vec{h}}_{t}+W_{{\overset{\leftarrow }{h}}_{t}}{\overset{\leftarrow }{h}}_{t}+b_{t}$$

Here, $W_{{\vec{h}}_{t}}$ and ${\vec{h}}_{t}$ denote the weight matrix and hidden state of the forward hidden layer at time t, respectively, while ${\overset{\leftarrow }{h}}_{t}$ and $W_{{\overset{\leftarrow }{h}}_{t}}$ represent the corresponding parameters for the backward hidden layer. $b_{t}$ denotes the bias term of the hidden state at time t.

The BiGRU architecture enhances the contextual feature extraction capabilities by integrating forward and backward hidden states. Furthermore, this paper proposes embedding a global attention mechanism after the BiGRU framework to generate a context vector c. Through dynamic weight learning, this mechanism enables cross-temporal feature interactions, thereby improving the representation capability of critical fault features and achieving superior global context integration.

**Figure 6:**
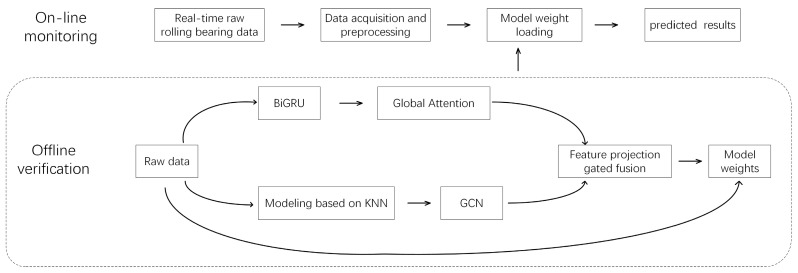
The overall training identification flowchart.

(3) Multi-Scale Heterogeneous Feature Fusion Module Based on Channel-Wise Attention Mechanism. In the feature fusion stage, this study proposes a cross-modal feature interaction framework based on a gated mechanism. Considering that different types of faults have varying sensitivities to spatial topological features and temporal dynamic features, this framework introduces a parameter-efficient gated unit to construct a nonlinear feature recombination mechanism to generate multimodal feature representations, which are then inputted into the classifier for fault category identification. On this basis, the system outputs a classification confidence vector and implements a dual-determination mechanism through the preset confidence threshold: when the confidence is higher than the threshold, it is determined as a known fault category; when it is lower than the threshold, the unknown fault identification function is activated. The standard process of online monitoring and offline training verification is shown in Figure 6.

Specifically, the gating mechanism dynamically adjusts interaction weights between graph convolutional features $z_{gcn}$ and attention-enhanced temporal features c, effectively capturing synergistic feature patterns of local graph structure anomalies and global spectral evolution. Firstly, the GCN features are mapped to the category space using Equation (16).

(16) $$z_{gcn}^{\prime }=W_{gcn}z_{gcn}+b_{gcn}$$

where $W_{gcn}$ denotes the projection matrix and $b_{gcn}$ represents the bias term. Additionally, perform the affine transformation of the context vector c according to Equation (17).

(17) $$c^{\prime }=W_{att}c+b_{att}$$

where $W_{att}$ denotes the parameter matrix. Finally, the gated weighted fusion is realized by Equation (18).

(18) $$f=W_{g}⊙z_{gcn}^{\prime }+\left(1-\sigma \left(W_{g}\left[z_{gcn}^{\prime };c^{\prime }\right]+b_{g}\right)\right)⊙c^{\prime }$$

Here, σ denotes the sigmoid activation function, and $W_{g}$ represents the trainable weight matrix. The fusion feature f outputs the original logits through the classifier and then transforms it into the mathematical form of probability distribution, as shown in Equation (19).

(19) $$P_{i,j}=Softmax\left(Wf+b\right)$$

where $P_{i,j}$ is the probability that the *i*th sample belongs to the JTH class, and the confidence is defined as the maximum probability value of each sample $Q_{i}=Max\;(P_{i,j})$. The unknown category is detected by a confidence threshold τ. If the confidence is lower than the threshold, it is predicted as an unknown category (class C) as detailed in Equation (20).

(20) $${\hat{y}}_{i}^{open}=\left\{\begin{array}{c} {\hat{y}}_{i}=\arg Max(P_{i,j}),Q_{i}\geq \tau \\ C,Q_{i}<\tau \end{array}\right.$$

This paper addresses the limitations of traditional bearing fault diagnosis methods in spatiotemporal feature co-modeling by proposing a cross-modal dual-channel diagnostic framework named ST-GABG, based on graph convolutional networks and bidirectional gated recurrent units (GCN-BiGRUs). An improved K-nearest neighbor algorithm is employed to construct graph topological structures, which, combined with three-level graph convolution incorporating post-average pooling, enables multi-order neighborhood information aggregation. Innovatively, a BiGRU-global attention fusion architecture is designed to enhance critical temporal node feature extraction through a dynamic weighting mechanism, while introducing a gating mechanism to achieve dynamic interactions between spatial topological features and temporal features. Systematic experiments are designed in subsequent chapters to comprehensively evaluate the framework’s performance from multiple dimensions, including the detection accuracy, robustness, and generalization capability.

## 4. Experiments

### 4.1. Experimental Setup and Training Evaluation Metrics

This paper conducts modeling analysis based on the Case Western Reserve University (CWRU) bearing fault dataset [38] and validates the ST-GABG model based on bearing datasets from Southeast University and Jiangnan University. The CWRU bearing test rig introduces single-point faults in test bearings through discharge machining with four different fault diameters: 0.007 inches, 0.014 inches, 0.021 inches, and 0.028 inches [39]. Due to incomplete measurement data for the 0.028-inch diameter condition, this study considers only experimental data from the other three fault sizes. In the CWRU bearing fault dataset, since the outer ring position is relatively fixed, outer race faults are further classified into three categories: 3 o’clock, 6 o’clock, and 12 o’clock positions. This paper employs vibration signals under normal operating conditions and multi-scale damage states as 12 Hz drive bearing faults occurring at 0 horsepower, specifically examining 0.007-inch, 0.014-inch, and 0.021-inch outer race faults (6 o’clock position). The Southeast University bearing dataset includes data types of healthy, rolling element fault, inner race fault, and combined inner-outer race fault under 20 Hz-0V and 30 Hz-3V load conditions. The Jiangnan University bearing dataset contains healthy, inner race fault, outer race fault, and rolling element fault types under variable rotational speeds (600 rpm, 800 rpm, and 1000 rpm). Each dataset comprises 120,000 fault records partitioned into training, testing, and validation sets at a 7:2:1 ratio. Detailed dataset fault information is presented in Table 1.

The experimental configuration is established as follows: all experiments are implemented on an AMD Ryzen 7 4800U processor with Radeon Graphics operating at 1.80 GHz and 16 GB RAM, utilizing Python 3.8 and the PyTorch-CPU 2.1.0 deep learning framework. This study systematically evaluates the BiGRU network depth through controlled variable analysis: with a fixed GCN layer count (three layers) and learning rate (0.003), performance testing across depths 1–5 reveals optimal accuracy at three BiGRU layers. Further depth increases the trigger gradient explosion risks and results in a 27% extension of the single-epoch training duration. Grid search optimization determines that an initial learning rate of 0.003 stabilizes the validation cross-entropy at ~0.004 by epoch 15, achieving 1.5× faster convergence versus the default (0.001). The implementation of a cosine annealing scheduler enables dynamic learning rate decay to 2.1 × 10^−6^, balancing aggressive parameter updates in early epochs with stable optimization in later stages. Parameter configurations are summarized in Table 2.

This study constructs a multi-dimensional evaluation framework comprising the cross-entropy loss, accuracy, F1 score, and macro-averaged recall. The model performance is systematically characterized through a combination of theoretical derivation and functional validation. The cross-entropy loss quantifies prediction bias based on information entropy theory, calculated in two steps during training: first, the output results are scaled between 0 and 1 using the Sigmoid function as formulated in Equation (21); subsequently, the final loss value is obtained via the negative log-likelihood loss function as defined in Equation (22).

(21) $$sigmoid\left(x\right)=\frac{1}{1+e^{-x}}$$

(22) $$h\left(p,q\right)=-\sum_{i=1}^{n}p\left(x_{i}\right)log(q(x_{i}))$$

After each training epoch, the recall is calculated using Equation (23).

(23) $$Recall=\frac{TP}{TP+FN}$$

*TP* and *FN* denote true positives and false negatives, respectively. Higher recall values and lower cross-entropy loss scores indicate superior model performance. The macro-averaged recall holds particular significance in deep learning multi-class classification tasks. This metric, which treats each class equally, is especially suitable for evaluating model recognition capabilities for minority classes under imbalanced class distributions, with its mathematical formulation derived in Equation (24).

(24) $$Macro-Recall=\frac{1}{m}\sum_{i=1}^{m}{Recall}_{i}$$

The F1 score comprehensively considers both model precision and recall. It is particularly applicable to scenarios with class imbalance, such as significant class distribution disparities between positive and negative samples, where it balances precision–recall trade-offs through harmonic mean computation. The mathematical formulation of the F1 score is presented in Equation (25):

(25) $$\mathrm{F1}\mathrm{Score}=\frac{2\times \mathrm{Precision}\times \mathrm{Recall}}{\mathrm{Precision}+\mathrm{Recall}}$$

In the performance evaluation of machine learning models, the systematic construction of multi-dimensional indicators has core value. This paper reflects the prediction uncertainty from the perspective of information theory through cross-entropy loss. The accuracy rate provides the overall performance benchmark. The F1 score balance precision rate is in contradiction with the recall rate, while the macro average recall rate focuses on the minority class recognition ability under class imbalance. It is particularly worth noting that in the process of optimizing deep learning models, this multi-dimensional index system can construct a diagnostic analysis graph. Through the collaborative verification among indicators, the precise location of model defects can be achieved, ultimately forming a closed-loop iterative mechanism of “error traceability—performance attribution—strategy optimization”, thereby systematically enhancing the generalization ability and decision-making reliability of the model.

### 4.2. Description of Comparison Methods

**Figure 7:**
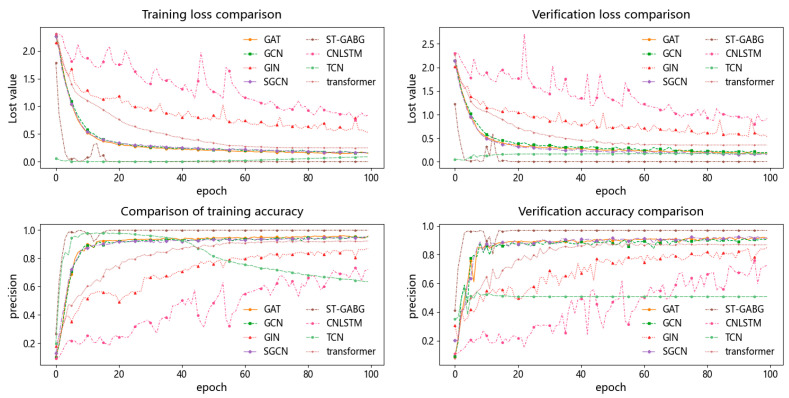
Comparison chart of loss accuracy rates of GCN, GAT, GIN, SGCN, and proposed method.

**Figure 8:**
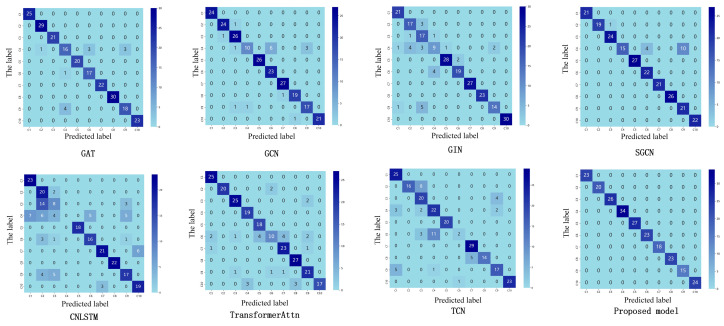
The three-dimensional confusion matrix of the proposed method and the comparison model.

To demonstrate the significant advantages of the proposed spatiotemporal joint diagnostic model (ST-GABG) in bearing fault diagnosis tasks, we compare it with various network models including the graph neural networks GAT, GCN, GIN, and SGCN and temporal models TCN, CNLSTM, and TransformerAttn. All methods are evaluated on identical datasets under consistent experimental environments—specific parameter configurations are detailed in Table A1. Diagnostic results across all methods are summarized in Table 3, leading to the following conclusions:

(1) Compared with traditional graph neural network models, ST-GABG demonstrates substantially superior performance. Specifically, while GAT and GCN achieve accuracy rates of 92.08% and 91.25%, respectively, ST-GABG attains a remarkable accuracy of 97.08%. This improvement stems from two critical innovations: first, by introducing a global attention mechanism that dynamically weights critical temporal steps after bidirectional GRU-based temporal feature extraction, ST-GABG effectively enhances spatiotemporal feature co-optimization. This approach not only reduces the training time by 9% but also improves the accuracy by 5% compared to the GAT model, which suffers from high computational complexity due to its reliance on single spatial attention mechanisms. Second, unlike GCN’s fixed neighborhood aggregation strategy, ST-GABG incorporates an improved KNN temporal-step joint encoding algorithm combined with a self-loop elimination weighted graph construction method. Through cross-validated graph parameter optimization, this approach achieves a 5.83% accuracy improvement. As shown in Figure 7, the proposed method can reach the verification loss convergence level more quickly and achieve a more stable loss curve. Combined with the early stopping strategy, it effectively reduces the training time. These results validate that the integration of bidirectional GRU temporal feature extraction with global attention dynamic weighting, synergistically combined with the improved K-NN algorithm, enables the effective fusion of temporal local–global features and complementary integration of spatiotemporal heterogeneous information.

(2) This study compares ST-GABG with three representative models: TCN, Transformer integrated with attention mechanisms, and CNN-LSTM fusion architectures. Specifically, TCN employs dilated convolutions to expand the receptive field for comprehensive global information capture, but suffers from substantial parameter overhead that increases the computational costs. In contrast, ST-GABG adopts a more lightweight global attention mechanism that significantly improves model efficiency. Compared to Transformer architectures that rely on self-attention for long-range dependency modeling but encounter computational complexity-induced inference latency, ST-GABG implements a cascaded design combining gated recurrent units with global attention. This approach effectively reduces the computational complexity while enhancing dynamic temporal feature capture through BiGRU’s memory update mechanism, achieving a 10.17% accuracy improvement. Furthermore, unlike CNN-LSTM’s stacked paradigm that sequentially extracts local features followed by sequence dependencies in separate phases, ST-GABG dynamically weights BiGRU hidden states via attention mechanisms to enable the deep fusion of local detailed features and global semantic information. Figure 8 visually demonstrates these performance advantages through 3D confusion matrix visualization.

### 4.3. The Ablation Experiments of the Proposed Method

**Figure 9:**
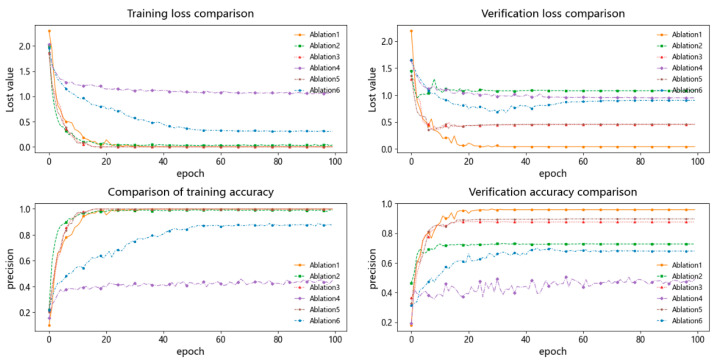
Ablation experiment loss and accuracy comparison chart.

The proposed multi-modal fusion methodology has demonstrated superior scene-adaptability diagnostic capabilities in preliminary experiments. This section further investigates the effectiveness of each functional module in the proposed model. Based on GCN, BiGRU, and global attention mechanisms, three ablation studies and three single-component control experiments are designed. Experimental results are summarized in Table 4, while Figure 9 compares validation loss curves across ablation groups. Through module combination analysis, this work quantitatively reveals the contributions of individual components to diagnostic performance. Specific quantitative findings lead to the following conclusions:

Compared with Ablation1 (BiGRU and Attention), Ablation2 (GCN and Attention), and Ablation3 (GCN and BiGRU) architectures, Ablation1 achieves 90.83% accuracy, significantly outperforming other configurations. Specifically, it demonstrates a 17.71% improvement over Ablation2 (without temporal modeling) and a 2.92% enhancement compared to Ablation3 (without attention mechanism). Results indicate that equipment degradation processes in industrial scenarios exhibit strong temporal dependencies, which pure spatial modeling struggles to capture. The BiGRU component synchronously captures historical evolution trends and potential future patterns of device states through forward/backward gated recurrent units, while the attention mechanism dynamically enhances critical feature representations and suppresses sensor acquisition noise interference. This synergistic integration of bidirectional temporal modeling and adaptive feature weighting substantially improves complex spatio-temporal pattern recognition capabilities.

By comparing the proposed method with the BiGRU and attention architecture, the introduction of graph topology structure modeling demonstrates superior feature representation capabilities. Specifically, the proposed approach achieves a 6.25% accuracy improvement over BiGRU and attention, which reveals the necessity of spatio-temporal heterogeneous feature fusion. This performance improvement arises from graph convolutional operations capturing spatial correlations between nodes, complementing BiGRU’s temporal dimension modeling. The significant performance gap between Ablation3 (without attention mechanism) and Ablation4 (without any enhancement modules) validates that sole reliance on single-module architectures fails to fully exploit latent information in spatio-temporal data, further substantiating the requirement for joint spatio-temporal reasoning in complex fault pattern analysis.

Through ablation studies comparing GCN and attention, GCN and BiGRU, BiGRU and attention, and the proposed method, the model’s ability to maintain high precision while significantly enhancing generalization capabilities in complex scenarios is validated. This advantage stems from the complementary modeling of spatio-temporal features through multi-modal fusion mechanisms. The average accuracy rates of the four architectures are 73.12%, 87.91%, 90.83%, and 97.08%, respectively, demonstrating the progressive performance gains achieved by integrating spatial–temporal heterogeneity and adaptive feature interaction.

### 4.4. Visualization of the Feature Extraction Results

**Figure 10:**
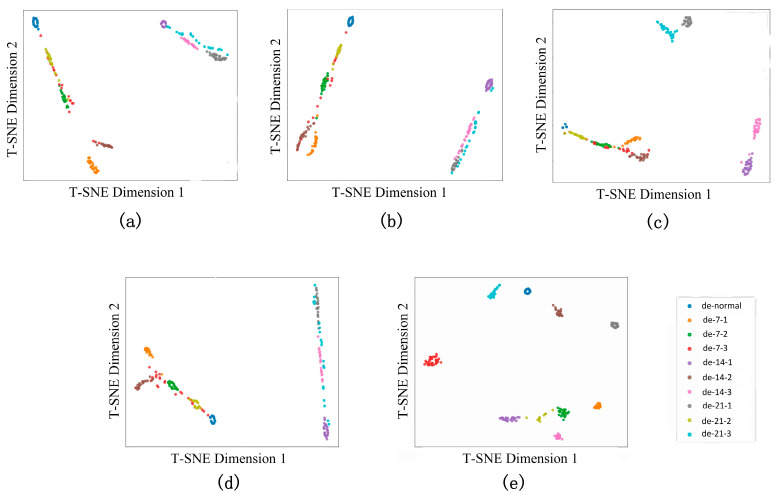
Visualization characteristics of the comparison method of T-SNE in multi-signal scenarios: (**a**) GAT; (**b**) GCN; (**c**) GIN; (**d**) SGCN; (**e**) proposed method.

**Figure 11:**
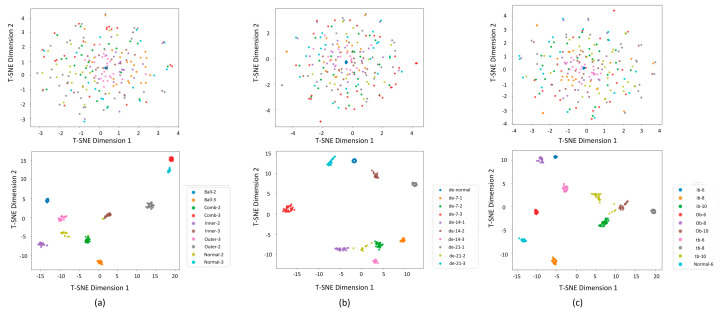
T-SNE scatter plots of the ST-GABG model under the bearing dataset of Southeast University (**a**), Case Western Reserve University bearing dataset (**b**), and Jiangnan University dataset (**c**), respectively.

To validate the deep feature extraction capability of the proposed model under rolling bearing signal-driven few-shot diagnosis scenarios, this study employs the t-distributed stochastic neighbor embedding (T-SNE) algorithm for nonlinear dimensionality reduction and visual analysis of high-dimensional feature spaces [40,41]. Class probability serves as one of the primary perspectives for evaluating model outcomes and identifying problematic samples [42]. As illustrated in Figure 10, conventional methods exhibit significant limitations with severe inter-class overlap and blurred decision boundaries in the projected space. The proposed model (subfigure e) demonstrates superior feature separability: specifically, intra-class compactness is significantly enhanced, inter-class discriminability is strengthened, and fault categories form independent clusters with explicit geometric margins; decision boundaries achieve higher clarity. This visualization validates the proposed model’s advantages in rolling bearing signal representation clustering from a geometric-topological perspective. Figure 11 demonstrates that ST-GABG exhibits excellent clustering representation capability and dataset-adaptability generalization performance across three bearing datasets from CWRU, Southeast University, and Jiangnan University. 

## 5. Conclusions

This study proposes a spatiotemporal joint modeling approach for bearing fault diagnosis, addressing critical challenges in traditional diagnostic methods related to multidimensional information fusion, dynamic feature capturing, and complex operating condition adaptation. Specifically, a graph topology structure based on a modified K-nearest neighbors (KNNs) algorithm is constructed to deeply explore spatial mutual information correlations among fault signals, enabling the extraction of diagnostic knowledge graphs with cross-condition generalizability. Furthermore, a global attention mechanism is embedded within bidirectional gated recurrent units (BiGRU), where the dynamic allocation of temporal feature weights enhances critical node perception capabilities, significantly improving the model’s precision in capturing long-range spatiotemporal context associations. A gated fusion module is ultimately designed to achieve the adaptive integration of dual-channel features, forming a spatiotemporal joint diagnostic architecture with strong interpretability. Through the experimental part, the effectiveness and universality of the model were fully verified on multiple public datasets. Notably, this work has two primary limitations: first, constrained by experimental conditions, the method has not yet been validated in real industrial scenarios; secondly, the current decision mechanism based on a fixed threshold is difficult to adapt to the change in the category distribution in dynamic scenarios, so we should explore the optimization mechanism of dynamic confidence thresholds.

## Figures and Tables

**Table 1:** Data set failure situation.

Dataset	Load	Fault Type
CWRU	12 Hertz 0 Mach	Normal
		0.007 inner, 0.014 inner, 0.021 inner
		0.007 ball, 0.014 ball, 0.021 ball
		0.007 outer, 0.014 outer, 0.021 outer
SEU	20 Hz-0V	Normal, ball, comb, inner, outer
	30 Hz-2V	Normal, ball, comb, inner, outer
JU	600 r/min	Normal, tb, ib, ob
	800 r/min	tb, ib, ob
	1000 r/min	tb, ib, ob

**Table 2:** Neural network hyperparameter configuration.

Optimum Model Parameters	Value
BiGRU Hidden Layer	3
GCN Hidden Layer	3
Fully Connected Layer	1
Learning Rate	0.003
Batch	32
Dropout Rate	0.9

**Table 3:** Diagnosis accuracies of all the methods.

Methods	Precision	F1 Score	Macro Average Recall Rate
GCN	91.25%	0.9742	0.9724
GIN	83.95%	0.8412	0.8212
GAT	92.08%	0.9356	0.9287
SGCN	92.70%	0.9399	0.9502
TransformAttn	86.91%	0.8798	0.8804
TCN	57.29%	0.5900	0.5800
CNLSTM	66.39%	0.7039	0.7286
**Proposed method**	**97.08%**	**0.9914**	**0.9906**

**Table 4:** Ablation study on performance comparison of GCN, BiGRU, and attention-based models.

Experiment Number	GCN	BiGRU	Attention	Precision	F1 Score	Macro Average Recall Rate
Ablation1		√	√	90.83%	0.9399	0.9385
Ablation2	√		√	73.12%	0.7253	0.7276
Ablation3	√	√		87.91%	0.9227	0.9233
Ablation4	√			50.62%	0.4850	0.5052
Ablation5		√		89.58%	0.9396	0.9337
Ablation6			√	70.00%	0.6652	0.6643
**Proposed method**	√	√	√	**97.08%**	**0.9914**	**0.9906**

## Data Availability

The data presented in this study are openly available in kyrr-y/BEARING.

## Appendix A

In the appendix, Table A1 lists the key hyperparameter Settings of the comparison methods in detail, and Table A2 abbreviation table shows the abbreviations in this paper for explanation.

**Table A1:** Core configuration of the comparison method.

Model	Learn Rate	Weight_Decay	Head/Kernel	Layer
GAT	0.0003		1	3
GCN	0.0003			3
GIN	0.0003			3
SGCN	0.0003		1	3
TCN	0.003		2	2
CNLSTM	0.003		3	2
TransformerAttn	1 × 10^−4^	1 × 10^−5^	2	2

**Table A2:** List of abbreviations.

Acronym	Fall Name
BiGRU	The Bidirectional Gated Recurrent Unit
GCN	Graph Convolutional Network
KNN	K-Nearest Neighbor
ST-GABG	The Proposed Spatio-Temporal Joint Diagnosis Model
CWRU	Case Western Reserve University
SEU	Southeast University
JU	Jiangnan University
TransformerAttn	Transformer Attention Fusion Architecture
CNLSTM	CNN and LSTM Fusion Architecture
T-SNE	T-Distributed Stochastic Neighbor Embedding
